# Dietary iron intake and anemia: food frequency questionnaire in patients with hereditary hemorrhagic telangiectasia

**DOI:** 10.1186/s13023-020-01554-x

**Published:** 2020-10-20

**Authors:** Federica Cavalcoli, Alberto Gandini, Irene Aglaia Matelloni, Francesca Catalano, Saverio Alicante, Guido Manfredi, Gianfranco Brambilla, Fernanda Menozzi, Federica Perolini, Egon Costi, Roberto Bertè, Elisabetta Buscarini

**Affiliations:** 1 Gastroenterology Department, HHT Reference Center, Maggiore Hospital, ASST Crema, Largo Ugo Dossena 2, 26013 Crema, Italy; 2 UOC Farmacia, Maggiore Hospital, ASST Crema, 26013 Crema, Italy

**Keywords:** Hereditary hemorrhagic telangiectasia, Nutrition, Iron deficiency, Epistaxis, Iron supplementation

## Abstract

**Background:**

Hereditary hemorrhagic telangiectasia (HHT) is a multisystemic inherited vascular disease characterized by a heterogeneous clinical presentation and prognosis. Dietary evaluation is relevant in HHT patients to provide adequate iron and nutrient intake. Additionally, different dietary items have been reported to precipitate epistaxis in this setting. Our primary aim was to investigate the dietary habits of HHT patients through a food-frequency questionnaire (FFQ) to evaluate the presence of precipitants and/or protective factors for epistaxis and the occurrence of possible dietary modifications. The secondary aims were to evaluate the nutritional intake of iron in HHT patients and the self-reported effect of iron treatments on epistaxis. From April 2018 to October 2018, a 138-item FFQ was provided to HHT patients followed up at the HHT Referral Center of Crema Maggiore Hospital. The relationship between food items and epistaxis was ascertained on a separate form. Daily iron intake was calculated to establish the mean iron content of food items reported in the FFQ.

**Results:**

One hundred forty-nine questionnaires were evaluated [72 females, median age 54 years (12–76). Overall, 26 (18%) patients reported dietary items that improved epistaxis (mostly blueberries and red fruits, green vegetables and legumes), while 38 (26%) reported some dietary items that exacerbated epistaxis (spices, chocolate, alcohol, strawberries and ginger). Dietary modifications were reported in up to 58% of cases. In HHT patients, the mean daily iron intake was 8.46 ± 2.78 mg, and no differences were observed in the iron intake of patients reporting a diet modification and those who did not.

**Conclusions:**

In the comprehensive management of HHT a healthy and balanced diet, with increased consumption of dietary items with a high iron content, should be encouraged.

## Introduction

Hereditary hemorrhagic telangiectasia (HHT) is a multisystemic inherited vascular disease characterized by telangiectasia and arteriovenous malformations (AVMs) in visceral and mucocutaneous vascular beds. HHT is estimated to affect approximately 85,000 patients in Europe [1]. However, because most health care providers have limited specific knowledge about this disease, the diagnosis may be delayed [1, 2]. To date, the diagnosis of HHT is based on clinical criteria, the Curaçao criteria [3] and evidence of pathogenic variants in known disease-related genes [4].

The clinical presentation and long-term prognosis of HHT are heterogeneous depending on the number, type and location of telangiectases and AVMs. In most patients, the main manifestation is recurrent bleeding from either nasal or gastrointestinal telangiectases that can lead to severe iron-deficiency anemia and require iron supplements and recurrent blood transfusions.

Epistaxis frequently occurs in HHT patients and significantly impacts their hemoglobin levels and quality of life. Most HHT patients experience nose bleeds at least once per week and up to 30% have epistaxis daily [5, 6].

Previous observations, based on international surveys, have suggested that several dietary items may precipitate epistaxis, and a recent study reported that HHT patients may spontaneously modify their diet to avoid food items perceived to exacerbate nosebleeds [7]. However, self-prescribed diets have been reported to be associated with an increased risk of vitamin and micronutrient deficiency in different gastrointestinal diseases [8]. In the setting of HHT, dietary modifications that may involuntarily reduce iron intake appear to be of concern.

Thus, we designed the IRONIA (IRON intake and anemia) study to:

11 investigate the dietary habits in a large series of HHT patients through a food-frequency questionnaire and evaluate the presence of precipitants and/or protective factors for epistaxis in the diet and the presence of possible dietary modifications to avoid foods that may precipitate epistaxis.
12 investigate the nutritional intake of iron in HHT patients compared with the estimated average requirement (AR) for the Italian population [9].

## Materials and methods

### Study design

From April 2018 to October 2018, all consecutive patients with a definitive diagnosis of HHT at the HHT Referral Center of Crema Maggiore Hospital were asked to complete a food-frequency questionnaire (FFQ); during the same period, the questionnaire was available online on the patient association website: www.hht.it. The dietary intake of participants was assessed using an anonymous semiquantitative FFQ. The study was approved by the local Ethical Committee, and informed consent was obtained from all participants.

The exclusion criteria were gastrointestinal disease-causing malabsorption (e.g., celiac disease and inflammatory bowel disease), renal failure and a history of eating disorders.

No specific dietary advices were provided to the patients before questionnaire administration in order to provide a faithful picture of patient’s dietary habits.

The semiquantitative FFQ comprised 138 items grouped into 7 food product groups. Data on the participant’s consumption frequency for a given serving of each food item on a weekly basis were collected. Examples of standard portion sizes were provided as a reference based on food models. Foods were grouped into beverages (including alcohol intake), dairy foods, meats and fish, cereal, fruits, vegetables, miscellaneous, and other dietary items. Additionally, participants were asked to provide information on their weekly use of iron supplements and/or iron medications (both oral and intravenous formulations). Demographic data and most recent values of hemoglobin, iron and ferritin were also investigated.

The relationship between specific food items and epistaxis was ascertained on a separate interview form based on the perception of the patients. Participants were asked to report aliments that seemed to improve nosebleeds in an unstructured survey. Dietary items previously reported to exacerbate epistaxis were presented on a separate sheet, and patients were also asked to report any additional item that they regarded as potentially precipitating nosebleeds.

Prior to analysis, the reported frequency for each food item was converted to daily intake and the portion sizes of the consumed food were converted to grams. Iron intake from single products was estimated using the following formula: iron intake (mg) = daily number of servings × typical iron content in one serving (as reported in the “L'indagine nazionale sui consumi alimentari in Italia: INRAN-SCAI 2005–06)" [10]. The total daily iron intake was obtained as the sum of the values of iron intake from all groups of products. The iron daily intake of HHT patients was compared with the average requirement (AR) for the Italian population as reported in the “Livelli di Assunzione di Riferimento di Nutrienti ed energia per la popolazione italiana” (LARN) [9]. Considering the different requirements in female patients of reproductive age, the results were divided according to gender and, for female participants, according to age > 50 years or < 50 years.

### Statistical analyses

Patients’ characteristics were analyzed by descriptive statistics. Continuous variables are reported as the means ± standard deviation; categorical variables are reported as counts (percentage). All data were tested for distribution normality via the Kolmogorov-Smirnoff test. Differences between groups were assessed using the Mann–Whitney and Kruskal–Wallis tests, as appropriate. Differences between percentages were evaluated by Fisher’s exact test and Student’s *t* test, as appropriate.

The relationships between variables were determined by Spearman’s coefficient. A *p* value < 0.05, two-sided, was considered statistically significant.

## Results

In the study period the FFQ was proposed to 72 patients attending the HHT Crema Center, of these 66 accepted to fill the questionnaire; 85 more questionnaires were filled by accessing the website of HHT Italian Foundations (which has 897 members*).* In total, 151 questionnaires were completed, two surveys were incomplete and were excluded from the final analysis. Thus, 149 questionnaires were evaluated. Among these, 72 participants were female and 77 were male, with a mean age of 53 ± 14 years.

Overall, 26 (18%) patients observed that some dietary items seemed to improve epistaxis. The most commonly reported protective aliments were blueberries and red fruits in 8 cases (31%), green vegetables in 5 (19%) and legumes in 4 (15%). Accordingly, 46% of these patients reported higher consumption of dietary items perceived to be protective. On the other hand, 38 patients (26%) reported that some dietary items could exacerbate/worsen nose bleeds. Among these, the most common were spices (especially chili pepper) in 13 (36%) cases, chocolate in 12 (36%), alcohol consumption in 10 (28%), strawberries in 3 (8%) and ginger in 3 (8%). A reduced intake of dietary items perceived to worsen nose bleeds was reported in 22 of 38 patients (58%). The data on dietary items affecting epistaxis are detailed in Table 1.

**Table 1:** Dietary items reported to affect nosebleeds in HHT patients

Dietary items reported to ameliorate-epistaxis; n (%)			Dietary items reported to worsen epistaxis; n (%)		
	Current Study	Finnamore et al. [7]		Current Study	Finnamore et al. [7]
Blueberries and reds fruits	8/149 (31)	5/260 (1.9)	Spices	13/149 (36)	**–**
Green vegetables	5/149 (19)	10/258 (3.7)	Chocolate	12/149 (36)	37/265 (14)
Legumes	4/149 (15)	4/258 (1.6)	Alcohol	10/149 (28)	**–**
Banana, melons	–	3/260 (1.2)	Strawberries	3/149 (8)	25/260 (9.6)
Other vegetables	3/149 (2)	4/264 (1.5)	Citrus fruits	–	21/262 (8)

In our population, 58 patients (32 females; mean age: 57 years) were prescribed an oral iron medication represented by ferrous sulfate (Fe++ 80–105 mg per tablet) and ferrous II) glycine sulfate complex (Fe++ 100 mg per tablet). Thirty-two (16 females; mean age: 55 years) were on parenteral iron supplementation, mostly ferric carboxymaltose (Fe++ 2 ml/100 mg). Additionally, 19 patients were taking iron-based nutritional supplements such as liposomal iron or sucrosomial iron. Among patients taking iron supplements/medication, 29 (26.6%) reported a reduction in epistaxis during the assumption, while 7 (6.4%) reported iron therapy worsened epistaxis. Of the latter, seven patients, two received both parenteral and oral iron therapy, one parenteral iron therapy and four oral iron.

In the HHT patients, the mean daily intake of iron was 8.46 ± 2.78 mg; the mean intake was 8.94 ± 2.85 mg/day in men and 8.66 ± mg/day and 7.98 ± mg/day in women aged < 50 years and > 50 years, respectively. Among the three groups, the main contributor to dietary iron was vegetables (32.1%), followed by cereals (31.3%) and meat, fish, meat and eggs (17.1%). The iron supply from different dietary items is reported in Table 2.

**Table 2:** Contribution of different food-items to dietary iron intake

	Iron daily intake mg; n (%)				
	Total	Male patients	Female patients	Female patients < 50 years	Female patients ≥ 50
Meat, fish, eggs	1.45 (17.1)	1.39 (15.6)	1.48 (18.1)	1.71 (19.7)	1.38 (17.3)
Dairy products	0.33 (3.9)	0.34 (3.8)	0.32 (3.9)	0.37 (4.2)	0.30 (3.8)
Cereals	2.65 (31.3)	2.79 (31.3)	2.61 (31.9)	2.90 (33.4)	2.49 (31.2)
Fruits and vegetables	2.71 (32.1)	3.05 (34.1)	2.45 (29.9)	2.45 (28.3)	2.45 (30.7)
Fat and oils	0.02 (0.2)	0.02 (0.3)	0.02 (0.2)	0.01 (0.2)	0.02 (0.3)
Others	0.57 (6.8)	0.65 (7.2)	0.48 (5.9)	0.59 (6.8)	0.44 (5.5)
Alcohol	0.74 (8.7)	0.69 (7.7)	0.82 (10)	0.63 (7.3)	0.89 (11.2)
Total	8.46 (100)	8.94 (100)	8.17 (100)	8.66 (100)	7.98 (100)

Comparison of the iron daily intake in HHT patients with the AR for the Italian population showed that the mean iron daily intake in male patients was significantly higher than the AR reported in the LARN (8.9 ± 2.9 mg/day vs 7 mg/day, *p* < 0.0005), the mean iron daily intake in female patients aged < 50 years was lower than the AR (8.7 ± 2.6 vs 10 mg/day, *p* 0.017) and the mean iron daily intake in female patients aged > 50 years was significantly higher than the AR (8.0 ± 2.7 mg/day vs 6.0 mg/day, *p* < 0.0005). An inadequate intake of iron was observed in 20.8% (16/77) of male patients, up to 70% (14/20) in female patients aged < 50 years and 18.7% (9/49) in female patients aged > 50 years.

We found no significant differences in dietary iron intake between HHT patients who reported modified diets and those who did not (8.2 ± 2.8 mg/day vs 8.4 ± 2.9 mg/day, *p* = 0.6739).

No differences were observed in the dietary iron intake of male patients and female patients aged > 50 years taking iron medication. However, female patients aged < 50 years not taking iron medication had a significantly higher dietary iron intake than those taking it (Table 3). Overall, in patients taking iron medication, iron intake (resulting from both diet and prescription) was significantly higher than the AR (p < 0.01).

**Table 3:** Comparison of the mean dietary iron intake between patients taking iron medications and those not taking it

	Male patients		Female patients < 50 years		Fermale patients ≥ 50 years	
	Iron medication	No medication	Iron medication	No medication	Iron medication	No medication
n	42	35	8	14	32	18
Mean ± ds (mg Fe/die)	8.5 ± 2.5	9.5 ± 3.2	7.0 ± 1.1	9.1 ± 2.7	8.0 ± 2.5	8.0 ± 2.8
*p* value	n.s		0.0272		n.s	

Data on the hemoglobin levels were available for 104 cases (51 male patients, 15 female patients < 50 years and 38 females > 50 years). In men, the mean hemoglobin value was 12.6 ± 2.5 g/dl; in this group, a diagnosis of anemia, defined as a hemoglobin value < 13 g/dl, was established in 31 patients (60.8%). For women aged < 50 years, the mean hemoglobin value was 13.3 ± 2.4 g/dl; a diagnosis of anemia, defined as hemoglobin values < 12 g/dl, was established in 5 patients (33.3%). In women aged > 50 years, the mean hemoglobin value was 12.1 ± 2.4 g/dl; 15 patients (39.5%) were anemic (hemoglobin values < 12 g/dl).

Data on blood iron and ferritin were available for 69 and 80 cases, respectively: the mean iron value was 44.2 ± 30 mcg/dl, while the mean ferritin was 27 ± 26 mcg/l.

## Discussion

Our data, from the first FFQ in a large series of Italian HHT patients, suggest that different dietary items may have an impact on occurrence of epistaxis.

However, although a possible association between diet and nose bleeds has been previously suggested, caution should certainly be taken in questionnaire data interpretation. In fact, the presence of possible pre-existing biases on the healthfulness or the detrimental effects of certain dietary items, which could have affected the responses to FFQ, has to be taken in consideration. Further studies on larger population are necessary to further elucidate the relationship between diet and epistaxis and inherent pathophysiological mechanisms.

With this caveat in mind, in our study the dietary items reported to exacerbate epistaxis were spices (especially chili pepper), chocolate, alcohol consumption, strawberries and ginger. Similarly a previous study also observed chocolate and strawberry to be perceived as precipitants for nose bleeds in 37/265 (14%) and in 25/256 HHT patients (9.6%), respectively [7]. Finnamore et al. [7] also reported citrus fruit intake to be possibly associated with nosebleed exacerbation, but this result has not been confirmed in the present study. Finally, no previous study has reported associations with alcohol, ginger and spice consumption. On the other hand, the present study suggested, for the first time, that some dietary items may have a protective effect on epistaxis in HHT patients. The most commonly reported protective aliments were blueberries and red fruits, green vegetables and legumes. Finnamore et al. investigated the presence of dietary items that could reduce nosebleeds in HHT patients, but the results were inconclusive, with only 3.73% and 2.32% of patients reporting green vegetables and meat or fish, respectively, as possibly helping epistaxis.

The pathophysiological mechanisms underlying the relationship between dietary items and nose bleeds have not been clarified. It can be hypothesized that foods rich in salicylates (such as chocolate, strawberries and alcohol) may facilitate the occurrence of epistaxis [11]. Additionally, dark chocolate has been reported to inhibit different enzymatic pathways involved in platelet activity [12]. However, dietary salicylates comprise several foods; thus, other mechanisms likely play a role in nosebleed onset. Finally, to date, few studies have addressed the possible protective effects of dietary items on epistaxis.

Of note and not unexpectedly in our study, a substantial proportion of patients reported that they modified their diet both avoiding items perceived to provoke nosebleeds or increasing the consumption of protective foods (58% and 46% of patients, respectively). Importantly, vitamin and micronutrient deficits (mostly calcium, folate and iron) are well-known consequences of self-prescribed and unbalanced exclusion diets in different gastrointestinal disorders [8, 13]. The importance of dietary restriction in the setting of HHT has not yet been investigated; however, inadequate iron intake is of special concern in these patients considering their predisposition to iron-deficiency anemia. Thus, a careful assessment of iron intake was performed in the present study. The mean dietary iron intake in HHT patients was 8.46 ± 2.78 mg, without a significant difference between HHT patients who reported modifying their diet to reduce/ameliorate epistaxis and patients who did not modify their diet. Further studies are necessary to evaluate the impact of dietary modifications on other vitamins and micronutrients.

The data on dietary iron intake showed that the mean dietary iron intake in male HHT patients and female HHT patients aged > 50 years was significantly higher than the AR. However, for female patients aged < 50 years, the mean dietary iron intake was lower than the AR, with inadequate intake in up to 70% of patients. Thus, a careful nutritional evaluation is particularly important for the subgroup of women < 50 years to optimize dietary iron intake.

Finally, iron medications and supplements are frequently prescribed for HHT patients, and in most cases, a protective effect on nosebleeds is observed. However, a recent report from Shovlin et al. suggested that iron treatments worsened epistaxis in a small subgroup of patients (4.8% of iron tablet users and 6.5% of iron infusion users) [14]. The data from our survey demonstrated a significant proportion of HHT patients (26%) reported taking iron medications to reduce nosebleeds; however, in 7 cases (6.4%), iron therapy seemed to worsen nosebleeds. These observations support the idea that a subgroup of HHT patients may be more prone to develop rapid changes in serum iron levels, leading to endothelial damage and, ultimately, hemorrhage. Therefore, in this subgroup of patients, it is of paramount importance to ensure a proper dietary iron intake.

Limitations of this study consist in the relatively small sample size for an epidemiological study. Further studies with larger sample size and external validation are needed to confirm our results. Moreover, we acknowledge the limitation of evaluating the association between epistaxis and dietary items based on patients’ perception, thus entailing a risk of placebo or nocebo effect and reducing the reproducibility of the results.

## Conclusion

In conclusion, the IRONIA study showed that a comprehensive evaluation of HHT patients would ideally include an evaluation of their dietary habits, though the feasibility of FFQ administration in a busy clinical setting has to be evaluated. A shortened form of the FFQ focusing only on the foods that appear to worsen or improve epistaxis may be proposed.

Current study does provide evidence that HHT patients may modify their diet to avoid food items perceived to provoke nosebleeds. Even though patients may perceive some dietary items to be associated with nose-bleeds occurrence, in absence of strong evidence-based data and considering the risk for micronutrients deficit, physician should suggest caution in modifying the diet to reduce bleeding symptoms. It is important to encourage HHT patients to follow a healthy and a balanced diet, increasing the consumption of foods with a high iron content and high iron bioavailability. Appropriate dietary habits appear particularly necessary in HHT women of childbearing age and in HHT patients who show worsened bleeding during iron treatment.

## Data Availability

The datasets used and/or analyzed during the current study are available from the corresponding author on reasonable request.

## Abbreviations

HHT: Hereditary hemorrhagic telangiectasia
FFQ: Food-frequency questionnaire
AR: Average requirement
IRONIA: IRON intake and anemia
LARN: Livelli di Assunzione di Riferimento di Nutrienti ed energia per la popolazione italiana
